# Challenges in international health financing and implications for the new pandemic fund

**DOI:** 10.1186/s12992-023-00999-6

**Published:** 2023-12-05

**Authors:** Garrett Wallace Brown, Natalie Rhodes, Blagovesta Tacheva, Rene Loewenson, Minahil Shahid, Francis Poitier

**Affiliations:** 1 School of Politics and International Studies (POLIS), University of Leeds, Leeds, LS2 9JT UK; 2 Training and Research Support Centre, Harare, Zimbabwe; 3 Centre for Policy and Impact in Global Health, Duke University, Durham, USA; 4 Global Health Institute, Duke University, Durham, USA; 5 Nuffield Centre for International Development and Health, University of Leeds, Leeds, UK

**Keywords:** Pandemic Fund, Pandemic preparedness and response, Global health financing, Global health governance, Health emergency preparedness

## Abstract

**Background:**

The failures of the international COVID-19 response highlighted key gaps in pandemic preparedness and response (PPR). The G20 and WHO have called for additional funding of $10.5 billion per year to adequately strengthen the global PPR architecture. In response to these calls, in 2022 the World Bank announced the launch of a new Financial Intermediary Fund (The Pandemic Fund) to catalyse this additional funding. However, there is considerable unclarity regarding the governance makeup and financial modalities of the Pandemic Fund, and divergence of opinion about whether the Fund has been successfully designed to respond to key challenges in global health financing.

**Methods/Results:**

The article outlines eight challenges associated with global health financing instruments and development aid for health within the global health literature. These include misaligned aid allocation; accountability; multistakeholder representation and participation; country ownership; donor coherency and fragmentation; transparency; power dynamics, and; anti-corruption. Using available information about the Pandemic Fund, the article positions the Pandemic Fund against these challenges to determine in what ways the financing instrument recognizes, addresses, partially addresses, or ignores them. The assessment argues that although the Pandemic Fund has adopted a few measures to recognise and address some of the challenges, overall, the Pandemic Fund has unclear policies in response to most of the challenges while leaving many unaddressed.

**Conclusion:**

It remains unclear how the Pandemic Fund is explicitly addressing challenges widely recognized in the global health financing literature. Moreover, there is evidence that the Pandemic Fund might be exacerbating these global financing challenges, thus raising questions about its potential efficacy, suitability, and chances of success. In response, this article offers four sets of policy recommendations for how the Pandemic Fund and the PPR financing architecture might respond more effectively to the identified challenges.

## Key questions

### What is already known?

- There are serious concerns about the emerging global pandemic preparedness and response (PPR) agenda and its ability to meet existing financing challenges.
- There remain significant questions about the design and functioning of the new Pandemic Fund and its ability to fulfil its remit to address PPR policy and financing shortfalls.
- The need to address these challenges / questions gain relevance considering failures associated with the COVID-19 pandemic.

### What are the new findings?

- We identify eight challenges related to international financing instruments and development aid for health within the existing literature on global health governance and financing.
- When measured against the Pandemic Fund, these challenges have been unclearly addressed or unaddressed within the current design and practices of the Fund.
- There is a need to rethink current PPR financing and its relationship to global health financing writ large, in order to address and respond to known PPR and global governance and financing challenges.

### What do the new findings imply?

- If new global PPR initiatives such as the Pandemic Fund are to be successful, then they must address the recognised challenges highlighted in this article. Without a more integrated and holistic approach to PPR financing and global health financing generally, any new health financing instrument will struggle to bring about significant improvements in PPR or global health outcomes more broadly.

## Introduction

The failures of the international COVID-19 response highlighted key gaps in pandemic preparedness and response (PPR) at global, regional, and national levels. In terms of financing related gaps only, among other things, these included a lack of emergency surge funding, inadequate adaptive capacities due to limited health system investments, insufficient ACT-A commitments, and the reallocation of health resources to COVID-19 activities [1–4]. As a result, calls are being made for additional funding of $10.5 billion per year to adequately strengthen the global PPR architecture. In response to these calls, in 2022 the World Bank announced the launch of a new Financial Intermediary Fund (FIF) for PPR to catalyse this additional funding. The Pandemic Fund aims to fill financing gaps, expand the ability of UN agencies and multilateral development banks (MDBs) to support capacity building at the country and regional level, and provide ‘greater agility at the global level through initial bridge financing, as other sources are mobilized’ [1]. Yet many technical and procedural aspects of the Pandemic Fund remain unclear, with divergent ideas about the role of the Pandemic Fund within wider PPR and DAH structures being proposed by the World Health Organisation (WHO), Group of Twenty (G20), World Bank, key stakeholders, and countries most affected by PPR deficits. As one example, the Pandemic Fund is not referenced in Articles 19 and 20 of the United Nations Pandemic Agreement (where PPR financing is meant to be addressed), suggesting a lack of global strategy regarding wider PPR financing and the specific role of the Pandemic Fund.

Moreover, there has been considerable divergence of opinion and lack of clarity regarding the governance makeup and financial modalities of the Pandemic Fund during its design phase [1, 2, 5]. Part of this emerges from the way the fund has been set up: the early stages of the Pandemic Fund have been described by some as following a ‘deeply retrograde, insular design’ characterized by a lack of wider stakeholder input and a dogmatic unwillingness to consider establishing an external multisectoral secretariat like that of the Global Fund to Fight AIDS, Tuberculosis and Malaria FIF [3]. Partly, the lack of clarity relates to a lack of transparency regarding how the Pandemic Fund’s design is being finalised and a lack of detail regarding its operational procedures. For example, in May 2023 the Pandemic Fund accepted proposals for US$350 million in a first round of funding to target surveillance, diagnostics and associated human resources (results released in July 2023). In terms of eligibility, only International Development Association (IDA) or International Bank for Reconstruction and Development (IBRD) countries were eligible to apply, as were regional entities and any of the 13 approved ‘Implementing Agencies’. The latter include development banks, UN agencies and major global health institutions. However, the guidance for proposals was ambiguous regarding what would be an appropriate and ‘catalytic co-investment’ in PPR, how best to align the Pandemic Fund’s mandatory use of 13 approved Implementor Agencies with national health strategies and institutions, how to meet requirements for the required ‘results-based’ approach to PPR and its alignment with national strategic purchasing procedures, and how and with what criteria proposals would be revised via the Pandemic Fund’s Technical Advisory Panel (TAP). As will be discussed below, the results of the first round of funding have only partially clarified some issues and, in many respects, have created greater ambiguity.

There are additional concerns of the Pandemic Fund falling short of the $10.5 billion estimated PPR funding required. Financing the Pandemic Fund might also lead to the diversion of aid already pledged for other health subsystems, further fragmenting an already complex PPR financing architecture [3, 4, 6].

These issues are not unique to the Pandemic Fund, but rather reflect broader challenges and already identified shortcomings facing international health financing instruments and development aid for health (DAH). The Pandemic Fund is borne out of the recognition and consequent need to overcome existing inefficiencies of a complex and fragmented health financing landscape [6]. It is the latest addition to the PPR financing landscape, an area already experiencing rapid growth in the level of funding and interested actors, adding to its complexity and fragmentation. An increasing number of actors and initiatives compete for limited financing, delivering often overlapping yet distinct mandates with the general theme of improving global health outcomes [7–9]. Although market logic is sometimes used to suggest that competition can spur effectiveness and innovation, it can also increase transaction costs, promote inefficiencies and inequities, and work against economies of scale [9]. In recent years there has been an increasing discussion of unequal power dynamics between global health actors, leading to the scrutiny of the global health financing architecture, the potentially distorting influence of external funder and agency priorities, the lack of country ownership of national initiatives, the limited participation of nonstate actors, and the lack of transparency within decision-making processes [10–15].

To better situate the Pandemic Fund within these debates, this article presents eight challenges often associated with international health financing instruments and DAH. It then assesses how these challenges are currently being addressed by the Pandemic Fund. In doing so, the analysis helps to inform current debates about the suitability of the emerging PPR agenda and discussions about the appropriate role, functioning, and integration of the new Pandemic Fund within a larger PPR and DAH agenda. As will be argued, it remains unclear how the Pandemic Fund is explicitly addressing the challenges we outline below, and which have had a more thorough treatment elsewhere [16]. The analysis suggests rather that the Pandemic Fund might be exacerbating these global financing challenges, thus raising questions about its potential efficacy, suitability, and chance of success if they are not addressed.

## Methods

This article is motivated by two aims: First, to outline key challenges / criticisms documented in the existing literature regarding PPR financing and global health financing more broadly. Second, to better understand the implications of these challenges for the Pandemic Fund and how it has positioned itself to address these challenges.

Key challenges in global health financing for summary were located through a series of rapid reviews [17, 18]. First, we conducted searches that included the terms ‘multilateral financing’, ‘international health financing’, ‘pandemic preparedness’, ‘multilateral governance’, and ‘global governance’. Key terms were constructed into search strings using Boolean Operators. Second, an initial search using Google Scholar was conducted, followed by an analysis of text words contained in the title and abstract of retrieved papers. These terms supplemented the preliminary terms outlined above. This refinement process allowed for inductive flexibilities and supplementary terms in the search strategy. A second search using a refined and focused term list was conducted using Google and Google Scholar to allow the capture of grey literature. In total, nine search rounds were conducted. Third, to capture challenges with international development financing instruments more broadly, given overlaps with health as well as the use of similar financial modalities across sectors, the review was expanded to also include literature pertaining to multilateral financing instruments. The aim of this exercise was not to present a systematic review and analysis of the existing literature, which would normally include the collation of empirical evidence from a tailored number of studies to focus on a particular research question [17]. Instead, the review sought to provide an overview of a large and diverse literature to identify main challenges and criticisms traditionally associated with global health financing and its multilateral funding instruments.

In doing so, we identified several criticisms within the existing literature on global health financing. The critiques pointed to thematic repetitions that coalesced around a few specifically important topics. The challenges that were the most frequently raised themes were shortlisted from a longer list of challenges identified. Eight were selected and summarised to provide criteria against which to assess the current governance and financing design of the Pandemic Fund. The selected challenges are some of the most widely recognised and well-rehearsed topics in the literature and therefore have relevance to any new financing instrument operating within the global health financing landscape. They include misaligned aid allocation; accountability; multistakeholder representation and participation; country ownership; donor coherency and fragmentation; transparency; power dynamics, and; anti-corruption.

After summarizing these key challenges, we then analysed the available information on the Pandemic Fund and emerging PPR financing to understand how these eight challenges are being recognized, addressed, partially addressed, or ignored by the Pandemic Fund. The underwriting motivation of this exercise is a normative one. Namely, it is based on the understanding that any new financial instrument such as the Pandemic Fund *should* at least attempt to respond to the key challenges outlined in this article and that any identified shortfalls merits critical reflection about the reforms required to do so.

## Results

The reviews allowed quick analysis of available and relevant literature to identify key challenges in PPR and international health financing. The eight challenges examined below are not an exhaustive representation of all governance-related challenges associated with international health financing and its instruments, but rather reflect well-rehearsed discussions in the existing academic and grey literature. It is also important to note that the challenges are cross-cutting, interconnected and reinforcing (e.g., compounding, moderating, and/or dependent), thus our presentation of each challenge individually is for ease of engagement and comprehension, and should not be understood as an attempt to oversimplify their interrelated complexity. An overview of each challenge is provided below.

### Misaligned aid allocation

Development aid for health (DAH) is often criticised for its lack of effective, efficient and/or equitable outcomes. A major component of this is that DAH is often ‘donor-driven’, symbolic of external “pet-projects” that are poorly integrated into national health strategies. As such, a common challenge facing the governance of global health financing instruments is the decision-making of how aid should be spent with multifarious implications. Overseas DAH is often used to further international funder and agency political goals and interests, with a preference for short-term political gains over longer-term global health goals [19, 20]. Additionally, external funders often prioritise funding of countries within their geographic area of influence and/or in areas of national interest [20–22].

The emergence of thematic trust funds, such as the Global Fund and GAVI, have been described as offering opportunities for ‘trojan multilateralism’ as they allow external funders to bypass existing allocation systems and influence institutional (e.g., the World Bank) priorities [13, 19], permitting a prioritisation of external funder needs. Furthermore, earmarked funding offers more control and oversight to external funders in alignment with their agendas [12, 23, 24]. There is often a misalignment between sector-specific global priorities and country-specific needs, with the imposition of external funder and agency ideas [10, 25]. A consequent side-lining of implementing country needs, results with distortion of country health sector priorities, vertical silos, diversion away from coordinated efforts for health system strengthening, and a mismatch between disease burden and funding priorities [26–29].

New financing instruments may also not generate new funding and divert funding away from their original purpose [7, 30, 31]. Funding can be substitutive, with DAH used to replace domestic spending on health, undermining investment from national governments [11]. Recently, pandemic PPR financing research found evidence indicating both Overseas Development Aid (ODA) and national budget reallocations away from other subsystems [4]. This diversionary impact appears in changing organisation priorities, as implementing agencies face challenges to align funding conditionalities and funder needs with their mission. In order to prioritise funding to continue their work, agencies move away from their original mandates and become more ‘donor-orientated’, also referred to as ‘mission creep’ [24, 32].

### Accountability

Governance structures of multisectoral funds can be ad-hoc and complex as they bring together many stakeholders with varying degrees of power and influence, complicating accountability [7, 33]. If decision-making roles are unclear, so too is who is accountable to whom [11, 34]. Increasingly there is a lack of effective accountability mechanisms, in particular multidirectional accountability downward to implementing countries and affected communities. For instance, in a review only two out of 43 multilateral organisations were rated as ‘strong’ on accountability [11, 19]. Yet, with overseas development aid receiving increased public scrutiny and widespread political distrust, external funders are under increased pressure to demonstrate ‘value for money’, ‘national interest’ and greater accountability to contributors [20, 23]. Earmarked funds can improve accountability on project-specific expenditures but demands focusing on financial accountability can discourage pooled funding or interventions with wider system mandates [20, 22, 24].

There is a risk of poor oversight and accountability in FIFs which lack in-country presence, rely upon fund partnership programmes, and are not covered by World Bank policies [13, 35]. This can make it hard for stakeholders to understand how programmes work, whether they further health goals, and can result in programmes being out of touch with local context and needs [35, 36]. A lack of clarity over the role of national governments in designing new initiatives hinders channels for public accountability [28]. In most cases, external funders do not have the same reporting requirements as do implementors and there is often no mechanism for implementors to hold funders to account [28, 34]. For example, FIFs do not fall under the mandate of the World Bank’s inspection panel, leaving no mechanism for implementing countries to raise concerns over funded initiatives [13].

The direction of accountability can be particularly complex for non-state actors such as philanthropic organisations, private corporations, and international NGOs. In access to medical technologies initiatives, such as COVAX, pharmaceutical companies are seen as key partners without clear accountability criteria defined for these actors [37]. The inclusion of civil society actors is often viewed as an important element for public accountability, yet it is not always clear who these actors are representing and to whom they are accountable. Most international CSOs or NGOs based in high-income countries would be formally accountable to their membership and funders and not necessarily to the populations they aim to support [38].

### Multistakeholder participation and representation

There often is poor representation of implementing countries in decision-making governing bodies and in discussion forums in international health financing [39]. Whilst some instruments, such as the Global Financing Facility, may increase participation of implementing countries this engagement could be seen as superficial, without participation in funding decisions [40]. Who originates engagement and participation processes has implications for interests and abilities to reform those processes. GAVI and Global Fund were created through coalition-formation processes whereas the World Bank was created by political elites. This has influenced and embedded particular governance structures and accountability mechanisms, fostering or hindering reform capacities [41].

Challenges to CSO participation and representation was a key discussion point in the literature. Numerous barriers hinder civil society engagement in international health financing governance activities in-country. The quality of civil society engagement across agencies and in-country engagement platforms varies, with some lacking procedures to facilitate meaningful engagement [8, 42–44]. Where formal structures are in place, few structural safeguards exist, resulting in multi-stakeholder platforms being dominated by governmental elites or hand selected CSOs [8, 45].

Poor engagement mechanisms are compounded by other factors hindering CSO participation. Such factors include limited experience of CSOs engaging in such forums, limited financial resources and time to join meetings, poor communication and awareness raising with civil society, and rushed processes with little notice [8, 14, 42–44, 46]. This can result in more resourced civil society actors (often large international NGOs) becoming civil society representatives in place of indigenous CSOs [42, 43]. Even where civil society representatives are engaged, therefore, questions about constituency representation remain [38]. This is often compounded by a lack of transparency in CSO selection processes [14, 42–44, 46]. Additionally, resource deficits for CSOs in-country can drive competition and distrust between organisations, disincentivising meaningful joint participation and collaboration due to a fear of losing funding [8, 42, 43].

### Country ownership

Country ownership of funded activities and policy decisions is important for the sustainability and effectiveness of projects in improving health, as recognised in the 2005 Paris Declaration on Aid Effectiveness [39]. Yet, achieving country ownership is impeded by external funder requirements to have oversight over how funds are being spent and their ability to control priorities through earmarked funding [11, 32]. Further, external funders are often heavily involved in project implementation, such as by ‘pushing’ technical assistance through international rather than national consultants/expertise [28].

Implementing countries are characteristically low-income, sometimes fragile states and dependent on external funding [43]. Yet, the complexities of international health financing architecture and the pressure of funder conditionalities place a high burden on implementing countries, making ownership difficult. Countries are overburdened with parallel and duplicative reporting requirements for external funders, creating high transaction and administrative costs [10, 29, 32, 35]. International funders and agencies often bypass existing national and sub-national mechanisms, governance structures, and coordination processes, making coordination of funding difficult for implementing countries and undermining country ownership [8, 10, 27, 28, 43]. That said, despite these challenges to country ownership, several cases exist of governments successfully retaining greater control over the direction and outcomes of international funded activities, particularly when funding is pooled between several funders with direct governing oversight by a multistakeholder steering committee chaired at the country level [32].

### External funder coherence and fragmentation

The international health financing architecture can be described as greatly complex, uncoordinated, inefficient and ineffective, consisting of a growing number of unaligned public, private and civil society actors creating a greater number of distinct yet overlapping funding instruments [20, 23, 32, 36]. Earmarked funding is a significant driver of incoherence in external funding, with this programme-specific funding model feeding into competition between agencies for resources, harming inter-agency coordination and strategic resource allocation [23, 24, 27]. Duplication and overlaps exist in the thematic and geographic foci of agencies, the types of activities being funded, and in creation of parallel national coordination structures [8, 24, 32].

Incoherence and fragmentation can have widespread consequences. It can make financial tracking, accountability, and program effectiveness difficult to assess, which can result in double-counting financial commitments and inflated program impact evaluations [47]. Complexity and fragmentation in the coordination of funds undermine potential synergies and economies of scale between funders and programmes of work. This is cited to reduce the effectiveness of health emergency response [6, 7].

Fragmentation and high levels of complexity can also be seen within financing organisations. For instance, international funding processes are reported as being scattered and decision-making on funding as often decentralised to the field or divided across different organisation departments [23]. ‘Super-PPPs’ (i.e., Public Private Partnerships) such as ACT-A and COVAX consist of particularly complex, fragmented governance structures, with key partners consisting of other PPPs, resulting in some actors being represented numerous times whilst obscuring the roles of others, undermining transparency and accountability [37].

### Transparency

The theme of transparency is often discussed within the context of governance, owing to the widespread implications of poor transparency, such as undermining trust between stakeholders, masking asymmetries in policy influence, and rendering reason-giving and programme accountability difficult. A lack of transparency makes independent research and evidence gathering difficult, posing a challenge in the pursuit of evidence-based policy. Governance structures can create uneven arrangements for information transparency. For example, local CSOs must rely upon personal relationships with government personnel or take significant efforts to gain information on how to engage with international funds [34, 42]. There is often opacity surrounding the organisational governance and decision-making processes of international financing instruments [34, 43]. Additionally, a 2017 analysis found that World Bank policies, reports and datasets did not meet required standards of transparency, with key information on policies, governance, and financial information often out-of-date, missing, or incomplete [13]. There has been a gap between commitment to versus actual levels of transparency across global health funding [26]. The uptick of PPPs in global health and thus the enhanced private sector involvement has negative implications for transparency in international health financing, particularly given requirements for confidentiality and secrecy of private corporate interests and activities, inviting wider reflection on the appropriateness of private funding in health financing and the risks of conflicts of interest [7, 36, 37, 48].

### Power dynamics

Despite a growing number of new funds and initiatives there remains a small group of external funders controlling a disproportionate amount of funding in global health financing. These include the United Kingdom, United States of America, European Union institutions and the Bill & Melinda Gates Foundation, thus representing a consolidation of influence from a small group of global elites [12, 13, 19, 20, 31]. Consequently, the penchant for top-down approaches by a small number of actors has been described as developmental paternalism which merely reiterates and reinforces existing global power dynamics [14, 37]. One example that reflects this ‘donor’ versus ‘recipient’ relationship, was the announcement of the Global Financing Facility whereby powerful states and the World Bank announced at a UN General Assembly that they were launching the new fund, with no evidence of meaningful participation in the design and selection of fund ‘beneficiaries’ [14]. Nevertheless, although many agencies and implementing countries rely on external funding from this small group of elites, creating program and operational dependencies, this can also be described as a co-dependent relationship. Funding organizations needing to justify budgets, impact and ‘value for money’ also depend upon ‘recipients’ through which to channel funds in-line with their mandates in a way that fulfils these expectations [20].

### Anti-corruption

Opportunities for corruption and fund misuse are created by the large amount of public and private funds being mobilised. Information and power asymmetries, poor transparency around decision-making and weak accountability mechanisms increase the risk of conflicts of interests and undue influence [7, 36, 49, 50]. Over recent years, international health financing organisations have increased efforts to mitigate corruption risks, largely through transparency and accountability mechanisms. However, these mechanisms can have large operating costs, and can shift resources away from health services, increasing administrative burdens on implementing countries, and hindering project implementation [49–52]. This is particularly the case with mechanisms such as performance-based financing (e.g. as associated with health FIFs) [53]. It is also difficult to provide a robust overview of anti-corruption governance in international health financing due to a lack of relevant literature. Evaluation of these organisations is difficult due to a lack of accepted standards and difficulties in measuring corruption, negatively impacting mitigating strategies [49–52].

## Discussion

The aforementioned challenges across the global health and international financing architecture should not come as a surprise to anyone studying global health financing. In many cases they are commonplace concerns with long traditions of critique. They are elaborated not necessarily to provide new insights, but to outline a series of general concerns and recognised challenges in global health financing. By doing so, these challenges are then used in this section to assess the emerging governance structure of the Pandemic Fund in order to explore the degree to which they are being addressed in this new instrument. Below we assess the Pandemic Fund against each of the challenges in turn.

### Misaligned aid allocation

As discussed, DAH is often criticised for its lack of effective, efficient and/or equitable outcomes that are poorly aligned with national health strategies. This creates vertical health siloes, which focus on singular coverage areas while diminishing efforts to strengthen integrated approaches, local buy-in, and ownership. Moreover, DAH conditionalities often reduce local control, flexibilities and needs-based responsiveness, undermining programme performance and population health outcomes. A focus on international aid priorities in the Pandemic Fund, such as for laboratories and surveillance, can leave vital and complementary system areas for prevention and impact management underfunded, weakening the whole chain of response, particularly for disadvantaged and underserved communities [54–56]. Since social and health inequality were risk factors of COVID-19 across different country settings, equity would be important for public health effectiveness [56, 57]. There is, however, no explicit guidance in the Pandemic Fund Governance Framework on how equity will be addressed in either the fund process, with reference to prioritised beneficiaries of programmes, health system or health-related features, or as a key deliverable to be assessed in any proposal. Finally, there is emerging evidence that there are ODA and national budget reallocations away from other health subsystems to PPR activities [4], further threatening to undercut health system strengthening efforts, while exacerbating universal health coverage vulnerabilities.

It is not clear whether challenges of misaligned programming have been suitability addressed by the new Pandemic Fund. What is clear is that eligibility for the first round of funding required countries to be eligible for IDA and IDBR funding and to demonstrate national co-investment and co-financing by at least one of the 13 approved implementing entities. Moreover, the Fund’s Technical Advisory Panel (TAP) scores proposals against a scorecard, with several questions on how well the proposal aligns with country and regional plans, as well as ‘country ownership’ [58]. In theory, the requirement for co-financing and national-level additionality should create opportunities for strategic alignment. However, the Pandemic Fund’s call for proposals did not provide guidance regarding the specific ways, standards, or best practices among global health agencies for co-financing or ensuring additionality. It also remains unclear whether other external sources can qualify as legitimate sources of additionality (such as bi-lateral aid) and whether demonstrated additionality should be measured by improved performance on preparedness, or merely on whether additional funding was secured. This raised concerns that the process relies heavily on the effectiveness of co-financing arrangements, the mechanisms of each implementing agency, and potentially other external funder conditionalities [59].

For the first round of funding, the World Bank received 179 applications from 133 countries with requests for over $2.5 billion in grants. The TAP, chaired by the WHO, selected 49 out of 135 eligible applications for recommended funding. The Governing Board then picked 19 out of these 49 [60]. In terms of programme requirements, the World Bank asserts that proposals will be reviewed by their ability to meet one or more of the following criteria, ‘strengthening comprehensive disease surveillance and early warning, laboratory systems, and human resources/public health workforce capacity’. Although the Pandemic Fund is clear that proposals must align with health security strategies, it is not clear on how far these preparedness activities should be aligned or integrated within wider health system strengthening efforts and/or broader national health strategies, both identified as key for long-term health security [15, 61–63]. Moreover, it remains unclear exactly how the Governing Board made their final decisions and with what balance of criteria. Although the TAP has a ‘score card’ with criteria for assessing proposals, there is not something similar for the Governing Board. Since the 179 individual proposals have not yet been released at the time of writing, nor have the scorecards for the 19 successful applications, it is not possible to determine common elements between successful and non-successful proposals.

The concern for better PPR alignment has been echoed by Fan and Smitham, who argue that the Pandemic Fund should not track whether government additionality relates to increases from a specific list of pandemic preparedness activities [59]. Instead, they argue, the Pandemic Fund should focus on the overall increases in government spending on public health activities relative to overall government spending, with this being inclusive of core functions of pandemic preparedness but also wider alignment to health system strengthening. This includes particularly those areas of health system functioning such as the integrated primary health care responses and community health systems that support equity in pandemic responses and health outcomes.

### Accountability

A crucial aspect for programme ownership, follow-through and effectiveness is multidirectional accountability in which principles of ‘partnership’ are embedded within DAH processes. External funders should rightfully know that their funds are ‘reaching the ground’ by their implementing partners and thus can be justified to taxpayers as having ‘value for money’, with this in everyone’s mutual interest. Correspondingly, implementing countries should rightfully have a genuine sense of partnership, where localized needs and control are being respectfully reflected in programme design, management, and evaluation. This multidirectional accountability is crucial since asymmetrical processes undermine trust, effective design, programme sustainability, and outcomes [15, 64]. Given the scale, complexity and urgency of the Pandemic Fund mandate, there is a real danger that a lack of appropriate accountability measures could fail to mitigate against unidirectional accountability (only upward to international funders and agencies), which endangers the possibility to break from DAH ‘business-as-usual’ [65].

### Multistakeholder participation and representation

An important takeaway from COVID-19 is the realisation that effective pandemic preparedness and response will require the coordination and input of multiple sectors and stakeholders. Multisectoral participation is necessary not only to align policies for more comprehensive and complementary PPR coverage but also to make sure that PPR activities align with local needs, wider policy and system goals, burdens of disease and upstream determinants. This speaks to the importance of public sector leadership in countries, and of a ‘partnership agenda’ in global health, as well as the facilitation of meaningful dialogue between stakeholders, particularly those with local expertise and implementation experience. This is recognised in the language of the Pandemic Fund [1, 58, 60, 66].

Yet, the Pandemic Fund looks as if it will be managed by an exclusionary group of the usual global funders and agencies. Although two CSOs were added to the Governing Board, this was largely in response to growing protests from key actors, after the main designs for the Pandemic Fund were complete, and only after much fanfare [67]. Now, it is unclear to what degree these CSO actors will be able to influence Pandemic Fund decisions and/or whether they will be co-opted into institutional power dynamics, as has happened with past instruments [68].

Community level consultation and engagement is purported to be fostered within the Pandemic Fund during its proposal stage, where the TAP scorecard provides a higher score for proposals that can demonstrate community engagement, input, and ‘co-creation’ [58]. Although the scorecard largely focuses on co-creation between global partners, this emphasis on the community level is found in at least two scoring areas. That said, the level of engagement and meaningfulness of community input will be scored on the narrative presented within the proposal, and it remains to be seen whether engagement will be largely tokenistic or genuine. What is clear is that failure to widen participation could result in ‘travelling models’ that are not fit for purpose and do not promote wider global health security.

### Country ownership

Global covenants have increasingly recognised the need for localised ownership and managerial autonomy in the design, implementation, and evaluation of DAH. Although the Pandemic Fund is promoting a ‘horizontally integrated approach’, the concern is that it is replicating traditional top-down approaches where international funders and agencies and high-income countries set the PPR agenda and control how it is implemented [69]. These critiques are not unfounded since CSOs gained seats and representation on the Pandemic Fund Board only after fierce worldwide criticism [67]. As critics suggest, the Pandemic Fund needs a better bottom-up approach that can take account of country and regional level needs [70]. There are concerns that the required use of 13 Implementation Entities embeds hierarchical structures, dependencies, and unidirectional accountability. One suggestion to broaden inclusiveness is to involve regional organisations in governance models, with the African Union Africa Centres for Disease Control and Prevention (CDC) recommended to represent African countries, with its links to regional organisations such as the East African Community, the East Central and Southern African Health Community, West African Health Organisation and Southern African Development Community, who have themselves played a role in supporting country health system capacities and responses to health security issues [15]. However, the African CDC and these partners have recently been left out as an Implementing Entity, despite the former being the coordinating agent for the continent’s disease control and prevention. Since the Africa CDC has only recently been upgraded to an autonomous public health agency under the African Union, their role as implementing partner may be clarified by African member state resolution on their relative disease control role viz. a viz. that of WHO AFRO. Within countries, while the Pandemic fund usefully includes investment in human resources, it is unclear whether this applies beyond specific technical personnel, to include the public sector leadership, co-ordination, and negotiation capacities that have been important for engaging and mobilizing private actors and domestic and international funders for pandemic responses, including for local production of health technologies [15, 54, 56, 71].

### External funder coherence and fragmentation

COVID-19 demonstrated that global, regional and national systems were unprepared and unable to suitably respond to the pandemic, with key factors including those identified from the review, particularly historic underfunding and policy fragmentation.

Regarding underfunding, the Pandemic Fund has a remit to generate the estimated $USD 10.5 billion annual funding requirement for PPR. However, as of August 2023, the Fund had thus far only secured financial commitments of $USD 1.9 billion from twenty-six donors, most of whom are G20 countries, the Bill & Melinda Gates Foundation, the Rockefeller Foundation and the Wellcome Trust [66, 72]. In terms of existing demand, the Pandemic Fund in the first round received 179 bids, equating to $USD 2.5 billion, while only committing $USD 338 million to the first round of financing, constituting a demand over eight times the allotted envelope [60]. This suggests that demand for financing is far greater than available capacities, which raises concerns about the Pandemic Fund’s ability to effectively and equitably govern PPR, while foreshowing criticisms that it is built to fail [73].

In terms of strategic financing, the Pandemic Fund has been critiqued for its heavy focus on ‘classic’ pandemic preparation such as surveillance, diagnostic capacity and related personnel and skills [74]. Yet learning from COVID-19 showed that although many of these science-based requirements for pandemic control were met, ‘the global management of the pandemic still failed in many respects’ [75]. This included the failure to ensure adequate global supplies and equitable distribution of key commodities for LMICs and the implementation of top-down approaches in some settings that failed to build on strengths of primary care and community health systems, especially given their important role in leveraging inter-sectoral action (markets, housing, transport and other infrastructures) for pandemic prevention [54, 56, 62, 76].

While the Pandemic Fund refers to ‘One health’ in areas covered, it is not clear if its ‘prediction of disease’ or ‘early detection’ will support upstream capacities and processes for this type of health impact assessment. Nor is it clear whether prediction and detection will also address upstream economic and wider sectoral determinants of health that increase risk or support improved links between technical dimensions of health security and wider health system goals. Although the World Bank has stated that other institutions and financing mechanisms are necessary to support PPR, and they may invest in these areas, the Pandemic Fund has not given them sufficient coverage, demonstrating that other capacities will play a secondary role in PPR and limiting links in building a more integrated systems response. For example, the Pandemic Fund does not contain financing for contingency funds, clear links with the WHO Contingency Fund for Emergencies, links to investments in community or primary health care systems or to One Health, intersectoral investments and funding mechanisms to prevent and manage the socioeconomic impacts of health emergencies, highlighting a gap in vital public health emergency management [55, 77].

Although the planned activities for the fund have been shown as necessary, they are more tailored to ‘strengthening’ and ‘building’, apparently thus geared toward pre-outbreak settings and without enough focus on response capacities. According to Boyce et al. it would require $124 USD billion over 5 years to reach ‘demonstrated capacity on each indicator of the Joint External Evaluation’, a key ‘element’ on the Pandemic Funds ‘results framework’ [77]. As a result, the Independent Panel for Pandemic Preparedness and Response has recommended a matched sum of $100 billion to be available for response efforts [77]. This implies that the Pandemic Fund is unlikely to be able to fund both efforts.

In relation to policy fragmentation, there is little indication of how the Pandemic Fund will interconnect and complement other PPR and global health initiatives. One way that the Pandemic Fund is attempting to increase coherence across international funders and agencies is by partnering with 13 Implementing Entities to channel funds, such as development banks, Global Fund and UNICEF, to complement already established financing mechanisms in LMICs. However, the Pandemic Fund lacks clarity on how funds will be split amongst these organisations, what level of required co-financing is appropriate, on their approach to implementation through these entities, nor on how far these entities will be expected to link with and engage continental and regional economic communities that include LMICs who play a role in supporting, harmonising, and providing capacities for country activities. These details are not available from the brief two-page summaries of the successful applications published by the Pandemic Fund [78]. It is also unclear whether entities such as the Global Fund are appropriate agents to implement PPR system reforms. What remains clear, considering the discussion above, is that not addressing these challenges threatens to render the Pandemic Fund yet another under-coordinated and fragmented institution that lacks meaningful political capacities, country level buy-in, or funds to prepare for the next pandemic [69].

### Transparency

The literature reviewed suggests that opacity in transparency mechanisms undermine stakeholders’ trust while also masking asymmetries in policy influence and subvert programme accountability. Widespread ambiguities in the original World Bank white paper signalled a lack of urgency regarding the importance of transparency for programme acceptance and buy-in, ignoring its key role in policy success. For example, the success of the Pandemic Fund will rely on its ability to generate new financing without competing for existing global health funding [4]. Yet, there is no strategy for how the World Bank will assure that existing global health financial commitments are not reallocated to the Pandemic Fund. Although the Pandemic Fund has launched a new financial tracker, it operates under the assumption that commitments are from ‘new sources’ and not reallocations. Moreover, there is a lack of new thinking in how to engage key shareholders toward effectively financing global public goods [4, 73, 77]. As argued by Glassman, this sort of strategy will be crucial to engage prospective funders in a way that can meet the PPR financing gap [73].

The Pandemic Fund has generally lacked transparency in how it prioritizes projects, how it balances between global and local initiatives, and in how it integrates criteria such as equity in funding decisions. The creation of an instrument for the TAP to score proposals [58] should allow for better consistency in decision-making and transparency in how funding decisions were made. Moreover, whereas earlier Pandemic Fund meetings were closed, the Secretariat now organises open meetings to increase transparency and has committed to consistently publish its minutes.

In terms of transparency about prioritizations, PPR is complex and involves global collective action, meaning investment needs a national and international perspective [79]. PPR projects will directly benefit countries and indirectly benefit the rest of the world, and vice versa. For example, surveillance for a low morbidity disease in one country may not directly benefit that country and there could be better uses for that money. Nevertheless, that same investment could be beneficial at the regional and global level to prevent a wider-scale outbreak [79]. A working group has been set up to consider prioritisation frameworks and resource allocation criteria for PPR, but developments have not yet been reported [79]. To increase transparency in decision making, this process will also have to consider governance concerns regarding bias towards external funders and high-income countries.

### Power dynamics

Power dynamics, or more accurately, power asymmetries within countries, and between states and non-state actors, particularly those at international level, are pervasive across global health governance. Through such international financing channels, powerful actors can have a tangible and concrete influence over the direction of agencies, national health system priorities in implementing countries and funding channels for selected implementing agencies [15, 80]. This creates a scenario whereby if an implementing country’s government disagrees with the international funder-selected priority area, they risk forgoing financial support. This may encourage adoption of external priorities that do not align well with local needs and priorities. Yet, foregoing needed funds will render larger financial shortfalls, with potential cascading effects. Currently there is little evidence that the Pandemic Fund will stray from this playbook, as it will likely only fund activities endorsed by powerful international agencies, funders and high-income countries, such as in a focus on capacities for surveillance and laboratories versus those for health and community system strengthening and distributed local production of health technologies, with poor or uncertain country ownership. Unsustainable and fragmented initiatives ultimately undermine meaningful strengthening of pandemic preparedness and response, while reinforcing and solidifying the relevancy and influence of the World Bank and traditional powers.

### Anti-corruption

Whilst the World Bank’s disbursement of funds may be vital to ensure financial solvency, experience also suggests that without proper transparency mechanisms, money can go unaccounted for and be misappropriated. This poses a threat that funds will be taken away from vital services with longer-term national implications, as corrupt actors enrich and entrench. Although anti-corruption mechanisms remained underspecified in the World Bank whitepaper, there is widespread understanding that robust monitoring and accountability mechanisms are needed. In response, a UN High-level meeting on PPR to discuss and develop an appropriate set of monitoring and accountability mechanisms for the Pandemic Fund was scheduled for September 20, 2023 [81]. Moreover, the World Bank published its Pandemic Fund Conflict of Interest Framework in March 2023, which is aimed to make sure the Fund operates with high standards of transparency and accountability’ [82].

Beyond mechanisms for public domain reporting and oversight by mandated national audit and parliamentary bodies, ongoing debates also need to address the risk of corruption in pandemic financing, whether in the form of bribes, embezzlement, fraudulent contracts, inflated pricing, insider trading or diversion of funds. Given the nature of the Pandemic Fund as a potential global institution dedicated to quickly respond to global threats (if given a role for surge funding), experience (from across countries at all income levels [83, 84]) suggests that the Pandemic Fund and the public bodies involved need to take and publicly report on appropriate measures, such as: open-contracting, pre-registration of suppliers, beneficial ownership information, freedom of-information acts, limits on conflicts of interest, oversight by public bodies, and sufficient investigative resources to bring cases promptly and to protect and reward whistle-blowers to avoid corruption [85].

### Limitations

We note several limitations in this research. First, there is a potential bias in the published literature reviewed on challenges in international health financing instruments in that it mostly comes from high-income countries or international institutions, and from international NGOs/CSOs. Importantly therefore, the eight challenges reviewed may not fully represent the perspectives and/or hierarchical prioritization in low-income countries or other traditionally underrepresented contexts. Moreover, only material written in English was reviewed, which excludes perspectives from non-English sources. Second, with the Pandemic Fund still in the process of designing and implementing its policies, many of the issues raised could still be ‘in process’ and may be addressed in due course. The assessment in this article is thus meant to be informative to help reflect on an emerging international health financing instrument at the centre of the PPR agenda. Third, there are changing and emerging lessons involving COVID-19 and PPR, with unclear and evolving evidence on longer term impacts of policy and measures. This assessment thus draws on current knowledge, and further assessment is needed as better evidence materialises. Lastly, effective PPR requires a holistic response beyond the actions of a single international health financing instrument. We have focused on the Pandemic Fund due to its key, and currently monopolizing, financing position in the PPR agenda. By doing so this understates the crucial need for a broader assessment of what a suitable and more holistic PPR global health architecture requires. This has received a more thoroughgoing treatment elsewhere [16]. In this paper we add Pandemic Fund insights to that wider assessment. Notwithstanding these limitations, we consider the themes selected and the analysis given in relation to the current Pandemic Fund design to have provided useful research evidence and information for ongoing policy dialogue.

## Conclusion

Greater levels of funding need to be mobilised for health security and linked to goals for universal health systems and healthy lives [4]. Whilst the Pandemic Fund is welcome in that regard as a new PPR-specific financing initiative, it is not necessarily an example of meaningful reform in light of the challenges above. Instead, the fund appears to inherit many of the governance challenges of other health financing instruments that came before it. Perhaps the biggest challenge is the need for political reform to contend with an asymmetrical global political economy that concentrates power in the hands of a few agencies, with limited transparency and accountability mechanisms. Doing so can bring to light and engage with the political processes that shape and influence the design and success of initiatives such as the Pandemic Fund. Health is political. Politics and power cannot be ignored. Exploring health with a global political economy lens can, therefore, inform how we can politicise PPR in the interest of fairness and efficiency.

In this vein, this paper endorses many of the normative and practical calls above for the better representation of interests (democratic principles – procedural justice) within the PPR agenda, and within global health policy more widely. Moreover, in this article we agree that there exist asymmetries in health outcomes and that PPR and global health financing ought to be measured against agreed equity commitments and distributions (distributive justice - fairness). Lastly, the findings support the notion that aid effectiveness requires a more comprehensive governance framework to align policies, promote defragmentation, enhance subsidiarity, and foster cooperation (efficiency).

There exist several global health governance recommendations that could help to address the PPR financing challenges highlighted in this article, particularly as they relate to the Pandemic Fund and its evolving remit. Four are particularly relevant.

First, current negotiations in the International Negotiating Body (INB CA+) for the Pandemic Agreement must make sure that the financing components of the Agreement address sustainable and equitable financing, misalignment, fragmentation, and siloed programming. This focus is necessary immediately, since Article 20, the main PPR financing provision, consists of only 423 highly generalized words. The Article lacks specific considerations for how the Agreement will provide a normative guide in coordinating PPR financing, nor in how PPR financing should interconnect with global health financing writ large. There is no mention of the Pandemic Fund and its role in relation to whether, and how, it will deliver main aspects of the Agreement, including International Health Regulations (IHRs) capacity building, emergency surge financing, and connections to the existing WHO Contingency Fund for Emergencies. This raises issues of coherence, as the rapid establishment of the Pandemic Fund has now created a degree of pathway dependency that the Agreement must now reconcile as any part of longer-term strategic financing. Any implications of entrenched fragmentation within the Agreement are far reaching, since the PPR governance and finance architecture already includes a new Medical Countermeasures Platform, a new International Pathogen Surveillance Network, and the new revised IHRs with its 300 + additions. All of these require financing, an issue that needs to be considered within the Pandemic Agreement. Currently it is unclear how the Agreement will act as an umbrella structure for these platforms, including for the Pandemic Fund. Thus, in relation to the challenges outlined in this article, the Agreement as it is currently presented does not represent a step change from the status quo.

As a result, one recommendation is to better use the opportunity of the Pandemic Agreement to align and coordinate the various financing demands associated with PPR and bring them into coherency. The negotiations are ongoing with a series of INB and sub-committee meetings scheduled up to the May 2024 Agreement deadline. One promising note is that a specific sub-committee (chaired by Brazil – with Canada, South Africa, and Malaysia as co-facilitators) for PPR financing was established at the INB in November 2023. There is also now recognition by state treasuries and finance ministries of the need to be more involved in discussions around the Agreement. As a minimum it is essential that the role of the Pandemic Fund is specified in these discussions, to clarify its role either as one of many instruments, or as the main PPR instrument, together with its goals and commitments.

Second, in terms of sustainable financing, a more sophisticated set of interventions could free up existing funds as well as raise new funds to help countries meet their IHRs and PPR targets. For example, a combination of sovereign debt cancelation [86], reducing capital flight and global tax abuses [87], and tackling illicit financial flows [88] would allow for greater in-country health investments, while reducing the level of dependency on external funders. In the case of the former, it is estimated that if the G20 and financial institutions had cancelled all external debt payments due in 2020 and 2021 by the 76 poorest countries, it would have liberated US$ 300 billion [89, 90]. In terms of raising new funds, there are arguments that a more reliable source of PPR financing could be a global tax on financial transactions, carbon, or airline flights to help fund global common goods such as PPR [91] or for dedicated funds to be reallocated from better financed defence budgets [92]. Funds, such as the Pandemic Fund, could also adapt their own internal governance mechanisms to better enable all countries, not just traditional external funders and influential stakeholders, to have a meaningful say in how funds are raised, managed and spent [93].

Third, increasing the scale and scope of civil society and community representation within global health governance as well as PPR financing instruments would help create better accountability, transparency, and overall legitimacy of the system. Although there is caution on the role of CSOs and NGOs in terms of their effectiveness, interests and representation of disadvantaged groups, there is also evidence to suggest that they can play a crucial role as part of a broader multisectoral approach, bringing voice from community or excluded groups often not heard in dialogue on global financing [45, 68, 94]. This is a debate that cannot be resolved here, yet it is important to note that it is a mainstay of public policy that effective collective action requires buy-in from stakeholders and that this buy-in will require inclusive procedural democracy [95].

There have been many proposals for how to increase representation of CSOs and NGOs [44]. What is lacking is the political will to reorganize global initiatives. This was certainly the case with the Pandemic Fund, which originally resisted CSO inclusion on the Governing Board, bending only once pressure mounted from key organizations and powerful states. Yet, the Pandemic Fund still excludes CSOs and NGOs from submitting proposals or acting as implementors. For now, the method for assuring CSO and NGO consultations falls on the will of submitting countries and remains a tick-box on the TAP score card.

Fourth, removing the barriers to more widely distributed production of health technologies (not just vaccines and medicines) such as diagnostics and equipment is also desirable and would require new policies to reduce constraints on intellectual property, to increase technology transfer, and to support local manufacturing and medical countermeasures to help reduce PPR costs and promote self-dependency. As demonstrated during COVID-19, the opposite is true, since pharmaceutical companies and several high-income countries were able to effectively hinder equitable access to vaccines, information, technologies, and medical countermeasures [96].

Again, these issues are currently being debated within the INB (specifically Articles 10, 11 and 12) and have become major bottlenecks in the Agreement, especially from LMICs. One potential mechanism for undoing gridlock could be to use more equitable financing commitments in Article 20, with a clearer role for the Pandemic Fund to deliver those commitments, to negotiate packages that allow losses in some areas to be offset from gains in other areas. This potential is already noted in Article 20, where an explicit link to Article 12 (Access and Beneficiary Sharing) is made, but where the details remain elusive. Meaningful investments in LMIC prioritized PPR areas, health systems strengthening, and PPR capacities via a better-defined Pandemic Fund could thus offer potential solutions for unlocking other parts of the Agreement. Moreover, Article 20 calls for establishing a new mechanism to finance the Agreement. This could be a reconfigured and expanded Pandemic Fund, or the establishment of an additional instrument(s), with each option having pluses and minuses. This needs to be decided or strongly steered by May 2024, marking a potential opportunity to take the discussed challenges into account.

However, as implied above, following through on these recommendations would require large-scale reform of the global health architecture beyond the Pandemic Fund itself, and a normative shift away from ‘donor’ driven policy to human-centred policy that aims to increase regional and country capacities and representation and better support human rights, invest in primary health care, and foster sustainable self-sufficiency [97], and strengthen health systems as a bulwark of health security [61]. Much of this starts with how health is financed [19]. If new global PPR initiatives and the Pandemic Fund are to be successful, then they must address the recognised challenges highlighted in this article and pursue structural reform. Without this, any new health financing instrument will struggle to bring about sustainable, equitable, and cost-effective improvements to health systems and health outcomes.

## Data Availability

All items reviewed are publicly available on-line. The datasets created and/or analysed during the current study are available from the corresponding author on reasonable request.

## Abbreviations

ACT-A: Access to COVID–19 Tools Accelerator
COVAX: COVID–19 Global Vaccines Access
COVID-19: Coronavirus disease 2019
CSO: Civil Society Organization
DAH: Development Aid for Health
FIF: Financial Intermediary Fund
GAVI: Global Alliance for Vaccines and Immunizations
G7: Group of Seven
G20: Group of Twenty
IBRD: International Bank for Reconstruction and Development
IDA: International Development Association
IHR: International Health Regulations
INB: International Negotiating Body for the Pandemic Agreement
LMIC: Low–and Low–Middle–Income Country
MDB: Multilateral Development Bank
NGO: Non–governmental Organization
ODA: Overseas Development Assistance
PPP: Private public partnership
TAP: Technical Advisory Panel
UACDC: Union of Africa Centre for Disease Control and Prevention
UN: United Nations
UNICEF: United Nations International Children’s Emergency Fund
WHO: World Health Organization
WHO AFRO: World Health Organization African Region
